# V2 hotspot optimized MVA vaccine expressing stabilized HIV-1 Clade C envelope Gp140 delays acquisition of heterologous Clade C Tier 2 challenges in Mamu-A*01 negative Rhesus Macaques

**DOI:** 10.3389/fimmu.2022.914969

**Published:** 2022-07-22

**Authors:** Tiffany M. Styles, Sailaja Gangadhara, Pradeep B. J. Reddy, Anusmita Sahoo, Ayalensh Shiferaw, Sarah Welbourn, Pamela A. Kozlowski, Cynthia A. Derdeyn, Vijayakumar Velu, Rama Rao Amara

**Affiliations:** ^1^ Emory Vaccine Center, Emory National Primate Research Center, Emory University, Atlanta, GA, United States; ^2^ Department of Microbiology, Immunology and Parasitology, Louisiana State University Health Sciences Center, New Orleans, LA, United States; ^3^ Department of Pathology and Laboratory Medicine, Emory School of Medicine, Emory University, Atlanta, GA, United States; ^4^ Department of Microbiology and Immunology, Emory School of Medicine, Emory University, Atlanta, GA, United States

**Keywords:** Deoxyribonucleic acid (DNA), Modified Vaccina Ankara (MVA), uncleaved pre-fusion optimized (UFO), Clade C Env, Simian-Human Immunodeficiency Viruses (SHIV) challenge, rhesus macaques

## Abstract

Stabilized HIV envelope (Env) trimeric protein immunogens have been shown to induce strong autologous neutralizing antibody response. However, there is limited data on the immunogenicity and efficacy of stabilized Env expressed by a viral vector-based immunogen. Here, we compared the immunogenicity and efficacy of two modified vaccinia Ankara (MVA) vaccines based on variable loop 2 hotspot (V2 HS) optimized C.1086 envelope (Env) sequences, one expressing the membrane anchored gp150 (MVA-150) and the other expressing soluble uncleaved pre-fusion optimized (UFO) gp140 trimer (MVA-UFO) in a DNA prime/MVA boost approach against heterologous tier 2 SHIV1157ipd3N4 intrarectal challenges in rhesus macaques (RMs). Both MVA vaccines also expressed SIVmac239 Gag and form virus-like particles. The DNA vaccine expressed SIVmac239 Gag, C.1086 gp160 Env and rhesus CD40L as a built-in adjuvant. Additionally, all immunizations were administered intradermally (ID) to reduce induction of vaccine-specific IFNγ+ CD4 T cell responses. Our results showed that both MVA-150 and MVA-UFO vaccines induce comparable Env specific IgG responses in serum and rectal secretions. The vaccine-induced serum antibody showed ADCC and ADCVI activities against the challenge virus. Comparison with a previous study that used similar immunogens *via* intramuscular route (IM) showed that ID immunizations induced markedly lower SHIV specific CD4 and CD8 T cell responses compared to IM immunizations. Following challenge, MVA-UFO vaccinated animals showed a significant delay in acquisition of SHIV1157ipd3N4 infection but only in Mamu-A*01 negative macaques with an estimated vaccine efficacy of 64% per exposure. The MVA-150 group also showed a trend (p=0.1) for delay in acquisition of SHIV infection with an estimated vaccine efficacy of 57%. The vaccine-induced IFNγ secreting CD8 T cell responses showed a direct association and CD4 T cells showed an inverse association with delay in acquisition of SHIV infection. These results demonstrated that both MVA-150 and MVA-UFO immunogens induce comparable humoral and cellular immunity and the latter provides marginally better protection against heterologous tier 2 SHIV infection. They also demonstrate that DNA/MVA vaccinations delivered by ID route induce better antibody and lower CD4 and CD8 T cell responses compared to IM.

## Introduction

Despite a concerted global effort to produce an efficacious vaccine against human immunodeficiency virus (HIV) for more than 30 years, a successful HIV vaccine is still a far reach. The RV144 Thai Trial, the only partially successful vaccine approach to date in humans induced only 31% protection after 3 years (1). However, from these experiences come the ability to learn from our previous shortcomings and generate a superior vaccine platform going forward. One platform that we pursued is the DNA prime and modified vaccinia Ankara (MVA) boost modality (DNA/MVA). This vaccine platform has been used for multiple viral or bacterial vaccines including HIV, SIV, TB, and SARS-CoV 2 among others (2–10). Our HIV/SIV vaccine constructs co-express full-length Gag Pr55 and form viral-like particles (VLPs) consisting of a Gag core coated with HIV envelope (Env) protein (2). These VLPs allow for the presentation of multimeric HIV Env protein, mimicking presentation seen on the surface of the viral virion, with the ultimate goal being to induce highly functional broadly cross-reactive antibody responses (2, 5).

A major hurdle has been developing an HIV Env immunogen capable of inducing this type of broadly effective antibody response against HIV. There have been vast improvements on the front of generating novel HIV Env immunogens in recent years. Currently there are three leading platforms for generating stabilized trimeric HIV env immunogens. These include the SOSIPs (11), native flexible linkers (NFLs) (12), and uncleaved pre-fusion optimized (UFOs) (13–15) platforms. SOSIPs aim to form trimers by creating a disulfide bond between cleaved gp120 and gp41domains (A501C and T605C), in conjunction with the I559P mutation with improved furin cleavage site sequence (REKR to RRRRRR) (16–20). NFL trimers are generated by replacing the furin cleavage site with a flexible glycine-serine peptide linker to inhibit cleavage induced conformational changes within the gp140/gp41 heterodimer as well as the I559P mutation (21). The UFO redesign builds on the NFL platform by incorporating a redesigned HR1 (22). To further improve the immunogenicity and efficacy of our DNA/MVA vaccine modality, we developed two MVA recombinants based on clade C Env C.1086 sequence one expressing gp150 (MVA-150) anchored on the membrane of Gag VLPs as in our traditional design and the other expressing soluble secreted gp140 UFO trimer protein (MVA-UFO) using a new design. The Env in MVA-UFO immunogen has a redesigned HR1 domain, a NFL fusing gp120 to the gp41 and additional stabilizing mutations at positions 64K, 433P, and 316W (22, 23). In addition, it expresses Gag VLPs without Env on them.

Post vaccine analytics from the RV144 Thai trial revealed antibody responses generated against the V2 hotspot (V2 HS) as one parameter that correlated with delayed acquisition (24). A similar vaccine trial conducted within our group (M22), which used C.1086 Env based immunogens for vaccination and a SHIV1157ipd3N4 challenge revealed that C.1086 Env-induced V2 HS directed antibodies were not capable of binding the V2 HS of the challenge virus. After careful analysis, it was discovered that the loss of binding to the SHIV1157ipd3N4 V2 HS was due to a tyrosine located at position 173 (2). This amino acid mismatch completely abolished binding of vaccine sera to the V2 HS of SHIV1157ipd3N4. Therefore, in the current study, the histidine at position 173 in C.1086 Env was mutated to tyrosine to match the clade C consensus sequence, which also mirrors the V2 HS of the challenge virus. In addition to the H173Y mutation, we introduced two other mutations (K166R and H170Q) into the V2 HS to make the V2 HS of the vaccine immunogen similar to the V2 HS of clade C consensus sequence in both DNA and MVA immunogens. Given the location of the V2 HS, at the apex of the trimer, antibodies that recognize this epitope can have the potential to perform effector mediated functions such as antibody dependent cellular cytotoxicity (ADCC) and antibody dependent cell mediated virus inhibition (ADCVI) which could prevent cells from becoming infected or mediate killing of cells once they become infected.

Another way this vaccine strategy differs from our traditional DNA/MVA vaccine strategy is the immunizations were given *via* intradermal (ID) rather than intramuscular (IM) route. IM immunization results in robust CD4 and CD8 T cell responses that play a critical role in controlling viral replication post infection (2). However, the downside to this robust activation is the increase in IFNγ specific CD4 T cells that have the potential to become viral targets during challenge (25). Parallel studies in the lab revealed that ID immunization of MVA following IM DNA prime reduce both the CD4 and CD8 T cell responses post vaccination (data not shown). With this in mind, we changed the route of both DNA and MVA from IM to ID. Given that the goal is to decrease the rate of acquisition, we forwent the added benefit of increased CD8 T cell responses to decrease CD4 T cells that could serve as potential viral targets.

To test the immunogenicity and efficacy of our new and V2 HS optimized clade C SHIV immunogens, we conducted the M23 macaque study. We paired our DNA construct, which expresses C.1086 gp160 HIV Env protein on the Gag VLPs with either MVA-150 (DM-150) or MVA-UFO (DM-UFO). The DNA/SHIV C.1086 construct also expressed a built-in rhesus CD40L adjuvant to provide additional priming of the CD8 T cell response (5, 26). These two vaccine regimens were compared based on immunogenicity and efficacy following repeat low dose intrarectal challenges with SHIV157ipd3N4. Vaccination with either vaccine regimen (DM-150 or DM-UFO) resulted in similar gp140 serum and rectal IgG and IgA responses as well as similar T cell responses in the blood, however, there was higher C.1086 V1V2, and C.1086 and SHIV1157ipd3N4 gp160 specific antibody response in the DM-150 group relative to DM-UFO. Interestingly, during the challenge we observed markedly slower acquisition of SHIV infection among the unvaccinated control Manu-A*01+ RMs compared to Mamu-A*01- RMs and hence we analyzed the protection by Mamu-A*01 status. Post challenge, the DM-UFO vaccinated RMs showed a significant delay in acquisition of infection relative to unvaccinated control RMs in Mamu-A*01- but not in Mamu-A*01+ RMs.

## Materials and methods

### Animals

Thirty young Indian male rhesus macaques (RMs) aged 2 years old from the Emory National Primate Research Center breeding colony were selected based on Mamu-A*01, Mamu-B*08, and Mamu-B*17 alleles and cared for according to the Animal Welfare Act and the National Institute of Health (NIH, Bethesda, MD) Guide for the Care and Use of Laboratory Animals using protocols approved by Emory University Institutional Animal Care and Use Committee (4). All animals were negative for Mamu-B08, and Mamu-B17 alleles and 12 animals were positive for the Mamu-A*01 allele.

### Immunogens

SHIV DNA encoding Gag from SIVmac239, clade C HIV C.1086 envelope (Env) with the K160N mutation (GenBank accession number FJ444399.1), Tat, Rev, and rhesus CD40L was constructed as previously described with some modifications (2). Briefly, PG1/SHIV1086C_2ACD40L was modified *via* site-directed mutagenesis at the V2 hotspot, the linear sequence directly proceeding the α4β7 binding site, to represent the consensus C sequence: K166R, H170Q, H173Y (24, 27). These mutations were confirmed by sequencing the entire Env insert as well as the Gag and CD40L inserts. To confirm proper insertion and expression, 293T cells were transfected with SHIV DNA for 48hrs and supernatant and cell lysate was probed with anti-Gag 2F12 (Cat no.2343, NIH AIDS Reagent Program), anti-HIV Env using ID6 antibody (Cat no. 1610, AIDS Reagent Program), and anti-CD40L antibody (Cat no. AF617, R&D Systems). Transfected cells were also stained with these same antibodies, in addition to PGT121 (Cat no. 12343, AIDS Reagent Program), PG16 (Cat no. 12150, AIDS Reagent Program), CH58 (Cat no. 12550, AIDS Reagent Program), CH59 (Cat no. 12551, AIDS Reagent Program), and PGT145 (Cat no. 12703, AIDS Reagent Program).

Two SHIV MVA’s were generated using MVA encoding Gag from SIVmac239 (kindly provided by Dr. B. Moss); clade C HIV C.1086 Envelope (Env) with the K160N mutation as previously described with some modifications (2). The V2 hotspot of Env was modified to represent the clade C consensus sequence as stated above for both SHIV MVA’s. The first SHIV MVA construct encoded HIV C.1086 gp150 Env protein (MVA-150) that contained amino acids 1-727. In conjunction with the SIV Gag, this SHIV MVA produces SHIV VLPs expressing Env on the surface of the VLP. The second SHIV MVA construct encoded HIV C.1086 gp140 secreted Env protein (MVA-UFO). In conjunction with SIV Gag VLP, this MVA expressed a secreted gp140 UFO trimer protein that has a modified HR-1 domain and a native flexible linker (NFL) fusing the gp120 and gp41 subunits together with stabilizing mutations (E64K, A433P, and A316W) that decrease binding to CD4 (28, 29). As with the SHIV DNA, DF-1 cells were infected with both SHIV MVA’s and protein expression was determined *via* western blot and flow cytometry as stated above. Since SHIV MVA-UFO does not express gp140 on the surface of Gag VLPs, ID6 antibody was used to verify gp140 expression intracellularly *via* flow and in the cellular supernatant using Westernblot analysis. The binding of various bnAbs and non-bnAbs to MVA expressed UFO protein was determined using ELISA by capturing the protein on a ConA coated plate followed by binding to respective antibodies as discussed previously (30). Each antibody was used at a concentration of 10μg/ml followed by detection using anti-human IgG at 1:1000 dilution.

### Immunization

Thirty RMs were divided into 3 groups of 10 animals each with comparable ages, weights, and Mamu-A*01 status prior to vaccination. Each group had 4 Mamu-A*01+ animals. Ten RMs did not receive any vaccinations and served as non-vaccinated controls. The remaining 20 RMs were vaccinated with 3mg of SHIV DNA at 0 and 8 weeks intradermally (ID). Since the vaccine was given ID, the 3mg dose of SHIV DNA was divided into 4 equal parts and given 2 doses/thigh for each animal. RMs were again divided into 2 groups of 10 monkeys/group and vaccinated ID with either MVA-150 or MVA-UFO at 1x10^8^ pfu at wks 16 and 32 post prime. This dose was also divided into 4 equal doses similarly to SHIV DNA and given 2 doses/thigh for each animal.

### SHIV challenge

Non-vaccinated controls and vaccinated RMs were challenged intrarectally with clade C tier 2 SHIV1157ipd3N4 as previously described (2, 31). Briefly, the virus was obtained from the NIH reagent repository (Catalogue number 11689, lot number 5 08/03/2012; contained 9.8 x 10^6^ TCID_50_/ml and 257 ng/ml of p27) and used at a 1:700 dilution, and 1ml for each challenge. Each animal received a maximum of 10 challenges or until productively infected on a weekly basis. An animal was considered positive for infection only after 2 consecutive positive viral load readouts greater than 60 copies/ml of plasma.

### Quantitation of SHIV RNA

SIV Gag copy numbers in plasma were determined as previously described (9). Briefly, total plasma RNA was extracted in duplicates, reverse transcribed and PCR amplified for quantitative real-time PCR analysis. The limit of detection for the assay was 40 copies/ml of plasma.

### Collection and processing of blood and rectal secretions

Peripheral blood mononuclear cells (PBMCs) were collected and isolated as previously described (9). Rectal secretions were collected using Weck-Cel sponges (Beaver Visitec, Waltham, MA) and eluted as previously described (32).

### T cell responses

Intracellular cytokine staining (ICS) was performed as previously described (2, 26) with some modifications. Briefly, PBMCs were stimulated with 1 µg/ml each of SIVmac239 Gag (125 peptides, NIH reagent resource Cat# 12364) or HIV C.1086 Env (212 peptides synthesized by Genemed Synthesis Inc) overlapping peptides pools with modification to the V2 hotspot (K166R, H170Q, H173Y) in the presence of 1 µg/ml anti-CD28 and anti-CD49d (BD Pharmingen, San Diego, CA) in RPMI 1640 complete media (containing 10% Fetal bovine serum, HEPES, Gentamycin, and Penicillin-Streptomycin). The HIV Env overlapping peptides (15mers overlapping by 11) was divided into two pools, Env1 and Env2. Env1 consists of the first 106 peptides (amino acids 1-435 of gp120) and Env2 consist of the last 106 peptides (remaining gp120 and gp41) of the HIV Env protein. After 1.5 hrs of stimulation at 37°C in 5% CO_2_, GolgiStop (0.5 μg/ml; BD Pharmingen) and Brefeldin A (0.5 μg/ml; BD Pharmingen) was added. After an additional 4.5 hrs incubation, the cells were placed at 4°C overnight. The following morning, cells were washed in FACS wash buffer (PBS with 2% FBS and 0.05% sodium azide) then surface stained with CD4-BV650 (clone L200; BD Pharmingen), anti-human CD8-AmCyan (clone SK1; BD Biosciences), and Live/Dead Fixable Near-IR APC-Cy7 stain (Invitrogen, CA) for 20 mins at 4°C. Cells were then washed, permeabilized with Cytofix/Cytoperm (BD Biosciences) for 25 mins at 4°C, and washed twice with Perm wash buffer (BD Biosciences). Intracellular staining was then done using a mixture of anti-human CD3-BV421 (clone SP34-2; BD Biosciences), anti-human interferon gamma (IFN-γ)-Alexa 700 (clone B27; BD Biosciences), anti-human tumor necrosis factor alpha (TNFα)-PE-CF594 (clone Mab11, BD Biosciences). Cells were washed with Perm wash buffer followed by FACS wash buffer, then resuspended in FACS wash buffer. Cells were acquired on a LSRII (BD Immunocytometry Systems, San Jose, CA) and analyzed using FlowJo software (Tree Star, Ashland, OR).

### Binding antibody responses

Anti-Env antibody responses were measured by enzyme linked immune sorbent assay (ELISA) using either C.1086 gp140 timer protein (K166R, H170Q, H173Y), murine leukemia virus gp70 scaffolded-V1V2 proteins (from Dr. Abraham Pinter, Rutgers Medical School), V3 peptide, or SHIV1157ipd3N4 gp120 (2). Briefly, plates were coated with antigen at 1 μg/ml in PBS and incubated overnight at 4°C. The following day, the plates were washed, blocked, and incubated for 1 hr with 3-fold dilutions of serum. For the standard, known concentrations of purified rhesus IgG (serially diluted) (NHP reagent resource) was captured using a 1:10,000 diltyion of anti-rhesus IgG (Rockland). Bound IgG was detected using peroxidase-conjugated anti-monkey IgG (Accurate Chemical and Scientific, Westbury, NY) and tetramethylbenzidine substrate (KPL, Gaithersburg, MD). The reaction was stopped by adding 100 μl of 2N H_2_SO_4_.

A customized Luminex binding antibody multiplex assay (BAMA) was used as previously described (2, 8, 33) to determine C.1086 gp140 trimer antibody responses in the rectal mucosa at the peak and prechallenge time points. Briefly, protein conjugated beads were mixed overnight at 4˚C with 5-fold dilutions of standard and serum or secretions. The standards were calibrated by ELISA (33) and consisted of pooled purified IgG or IgG-depleted serum (for IgA assays) from SHIV vaccinated and infected macaques (34). Beads were alternatively washed and mixed for 30 mins with 100µl of 20µg/ml biotinylated anti-monkey IgG or IgA (Rockland Immunochemicals, Pottstown, PA) followed by 1:400 neutravidin-phycoerythrin (SouthernBiotech, Birmingham, AL). After measurement of fluorescence in a Bioplex 200 (BioRad, Hercules, CA), the concentrations of antibody were interpolated from standard curves. Antibody concentrations in secretions were divided by the total IgG or IgA concentrations to obtain the specific activity. Total IgG and IgA were measured by ELISA using goat anti-monkey IgG or IgA antibodies (Rockland) as described (32).

### Antibody Dependent Cellular Cytotoxicity (ADCC)

The ADCC assay was performed as previously described (2, 35). Briefly, 1x10^6^ CCR5+ CEM NK^r^ cells encoding a tat-inducible luciferase promoter (kindly provided by Dr. David Evans, University of Wisconsin at Madison) were infected through spinoculation with 200 μL of the SHIV1157ipd3N4 challenge stock virus for 3 hrs at 1500xg at 25°C. Infection proceeded for 4 days. On day 3, SIV Gag protein levels were checked *via* flow cytometry with 2F12 antibody. On day 4, infected targets were incubated with serum (1:100 dil) or monoclonal antibodies and rhesus CD16 expressing KHYG1 NK effector cells (from Dr. David Evans) at a 10:1 effector:target ratio for 8 hrs. After incubation, 150 μl volume of the cell mixture was then added to 50 μl of BriteLite Plus luciferase substrate reagent in a black 96 well plate (both from Perkin Elmer, Duluth, GA). Luciferase activity was measured after 2 mins. ADCC activity was calculated as the percent reduction in luciferase when compared to effector and target cells alone.

### Antibody Dependent Cell Mediated Virus Inhibition (ADCVI)

This assay was performed as previously described with some modifications (36). Briefly, on day 1, CCR5+ CEM NK^r^ cells were spinoculated at 1500xg for 3 hrs with SHIV1157ipd3N4 virus at 31 TCID_50_/mL. On day 2 of the assay, cryopreserved PBMCs were thawed, washed, and counted. Cells were added to a V-bottom plate (Corning, New York, NY) at a concentration of 1x10^5^ cells/well and allowed to rest overnight. On day 3, infected CCR5+ CEM NK^r^ cells were washed 3x with RPMI while serum was filter sterilized at a 1:25 dilution. Next 50μL of 1:25 diluted serum was incubated for 2 hrs with 1x10^4^ infected CCR5+ CEM NK^r^ cells. After incubation, serum and infected CCR5+ CEM NK^r^ cells were added to PBMCs. PGT121 and EM4C04 served at positive and negative controls respectively. Five days post incubation, cells were washed 2x and fresh media added to all wells. On day 7 post incubation, plates were spun at 1500 rpm for 5 mins and the supernatant was harvested and frozen until p27 Gag ELISAs could be performed

### SIV gag p27 ELISA

High binding ELISA plates (ThermoFisher, Waltham, MA) were coated at 1μg/mL with goat anti-mouse IgG2b (Southern Biotech, Birmingham, AL) overnight at 4°C. The next day, plates were washed 6x with 0.05% PBS-tween (PBST) and blocked with 1% BSA in PBST for 30 minutes at RT. Mouse anti-p27 2F12 antibody (AIDS reagent Program), at 1μg/mL, was added to plates and incubated for 1 hr at 37°C. After washing plates 6x, p27 standard (Immune Technologies) and culture medium treated with 0.5% Triton X-100 detergent (Sigma) to lyse virus particles were added in 3-fold dilutions and allowed to incubate for 1.5 hr at 37°C. Plates were washed and biotinylated SIV IG diluted 1:1000 (prepared in the laboratory) and added to each well. After 1 hr at 37°C, plates were washed, and 1:4000 neutralite-avidin peroxidase (Southern Biotech) was added. After 30 min at RT in the dark, bound IgG was detected using tetramethylbenzidine substrate (KPL, Gaithersburg, MD). The reaction was stopped by adding 100 μl of 2N H_2_SO_4_.

### TZM-bl neutralization assay

Envelope gp160 expression constructs 1086.C K160N and SHIV 1157ipd3N4 were described previously (37). Clade C consensus hotspot mutations (K166R, H170Q, H173Y) were inserted into the 1086.C K160N plasmid as follows. A fragment containing the 1086.C K160N Env expression plasmid nucleotide sequence from BamH1 (in vector) to PflM1 (after 1086.C aa 171 (HXB2 aa 185)) was synthesized to include the V2 hotspot changes (IDT) and inserted *via* InFusion into the parental plasmid. The final construct was verified by nucleotide sequencing.

Neutralization was measured using serially diluted, heat-inactivated immunized RMs serum, in the TZM-bl assay as previously described, using cells plated one day prior to the assay (37–41). In brief, Env pseudovirus was generated by transfecting the Env-expressing plasmid DNA alongside the HIV-1 SG3ΔEnv proviral backbone DNA into 293T cells, using the Fugene HD reagent as recommended (Promega). Pseudovirus stocks were collected from the 293T cell supernatants at 48 hours after transfection, clarified by centrifugation, divided into small volumes, and frozen at –80°C. 2,000 IU of each titered Env pseudovirus (in DMEM with ∼3.5% (vol/vol) FBS (Hyclone) and 40 mg/ml DEAE-dextran) was mixed with five-fold serial dilutions of heat-inactivated serum and assayed for inhibition using the TZM-bl indicator cell line, with luciferase as the readout. At 48 h post-infection, the cells were lysed and luciferase activity was measured using a BioTek Cytation3 multimode microplate reader. The average background luminescence from a series of uninfected wells was subtracted from each experimental well. Experimental wells were compared against virus without a test reagent (100% infectivity). All assays contained duplicate wells and were repeated at least once independently. Neutralization ID_50_ or IC_50_ titer values were calculated in Graphpad Prism using the dose–response inhibition analysis function with variable slope, log-transformed x values, and normalized y values.

### Statistical analysis

Graph-Pad Prism v8.0 was used to perform all nonparametric two-tailed statistical analyses. For statistical comparisons between DM-150 and DM-UFO a non-parametric test was utilized. For comparisons between time points within the same group, the Wilcoxon matched pairs test was used. The Spearman rank test was used for correlations. Statistical significance was defined as a P value of < 0.05.

## Results

### V2 hotspot (HS) modified DNA and MVA immunogens

**Figure 1:**
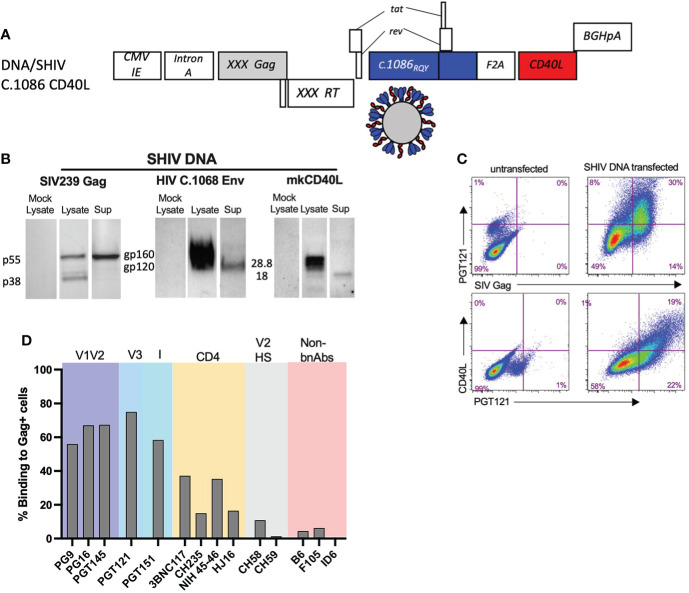
Characterization of SHIV DNA. **(A)** Schematic of SHIV DNA construct. **(B)** Western blot analysis of SIV Gag, HIV C.1086 Env, and Rhesus CD40L in the lysate and supernatant of transfected 293 T cells. **(C)** Flow cytometry of 293 transfected cells depicting the surface co-expression of HIV Env and rhesus CD40L on SIV Gag positive cells **(D)** Surface binding of bNAbs and non-bNAbs to SIV Gag positive DNA transfected cells.

SHIV DNA and MVA immunogens were constructed as previously described with some modifications (2). We modified the Env V2 HS of both the SHIV DNA and MVA immunogens at 3 key positions (K168R, H170Q, and H173Y) that contributed to the stabilization of the HIV envelope trimer and induction of broader anti-V1V2 scaffold specific response in rabbits (30). The DNA/SHIV C.1086 CD40L (will be referred to as DNA/SHIV-40L here after) expressed SIVmac239 Gag, reverse transcriptase (RT), and protease (Prt), and HIV Env gp160, Tat, and Rev from C.1086, with macaque CD40L as a molecular adjuvant (**Figure 1A**) (42). We confirmed the expression of Gag (pr55 and p38), Env (gp120 and gp160), and CD40L using western blot analysis and their co-expression *via* flow cytometry analysis (**Figures 1B, C**). As expected from VLP formation, the Gag pr55, Env gp120 and CD40L proteins were also detected in the cell supernatants (**Figure 1B**). A panel of monoclonal antibodies (mAbs), including some broadly neutralizing antibodies (bNAbs) and non-neutralizing antibodies were utilized to characterize the trimeric nature of Env expression on the surface of DNA/SHIV-40L transfected cells. Similar to what we observed in our previous M22 study, we observed strong binding (50-70% positive cells) with bNAbs directed against V1V2, V3 and Interface (I), and intermediate binding (15-40% positive cells) to CD4 binding site directed bNAbs and poor binding to (0-15% positive cells) non-bNAbs, including B6 and F106. We included two V2 HS specific antibodies (CH58 and CH59), which have varying sensitivity to the histidine at position 173 (2, 43). CH58, which is more promiscuous than CH59, bound at a lower level to DNA/SHIV C.1086. However, CH59, which is highly sensitive to the histidine at position 173, did not recognize envelope expressed by DNA/SHIV C.1086. Along with sequence analysis, these data confirmed the presence of a tyrosine at 173 (**Figure 1D**). These data indicated that the majority of envelope expressed on the cell surface was present in the trimeric form.

**Figure 2:**
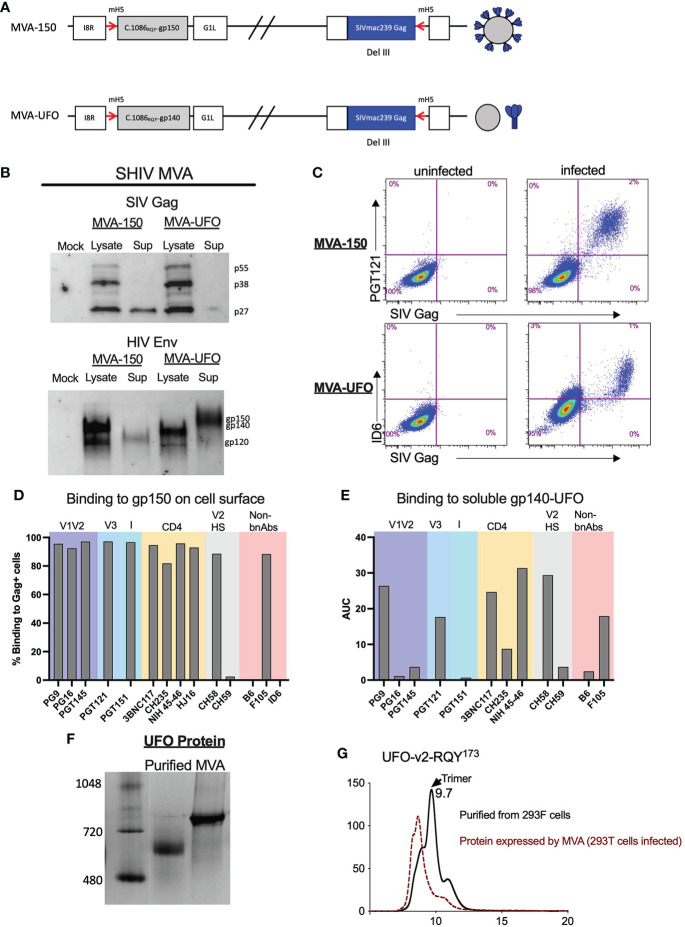
Characterization of SHIV MVA-150 and MVA-UFO. **(A)** Schematic of SHIV MVA gp150 and gp140 constructs. **(B)** Western blot analysis of SIV Gag and HIV C.1086 Env in the lysate and supernatant of infected cells. **(C)** Flow cytometry of DF-1 infected cells depicting the surface co-expression of HIV Env on SIV Gag positive cells. **(D)** Surface binding of bNAbs and non-bNAbs to SIV Gag positive MVA infected cells. **(E)** bNAbs and non-bNAbs binding to secreted C.1086 gp140_UFO_ protein *via* ConA ELISA. **(F)** Western blot analysis of purified gp140 C.1086 UFO protein and MVA secreted gp140 C.1086 UFO protein. **(G)** SEC profile of purified and MVA secreted C.1086 UFO protein.

We constructed two MVA recombinants, one expressing the C.1086 gp150 (MVA-150) that is anchored on the cell membrane and the second expressing stabilized gp140 UFO trimer (MVA-UFO) that was secreted. Both MVAs co-expressed SIVmac239 Gag Pr55, RT and Prt, and expected to form Gag VLPs except that only with MVA-150 the VLPs incorporate gp150 on the VLP membrane (**Figure 2A**). Protein expression for MVA-150 and MVA-UFO was determined as previously described (2). Similar to DNA/SHIV-40L, SIV Gag and HIV Env protein expression was confirmed *via* western blot analysis and flow cytometry (**Figures 2B, C**). The same panel of bNAbs and non-bNAbs were used to characterize Env expression on the surface of MVA-150 infected cells. Similar to what was observed for DNA/SHIV C.1086, V1V2, V3, I, and CD4 binding site directed antibodies bound extremely well to Env on the surface of MVA-150 infected cells. We also observed strong binding of CH58 to MVA infected cells despite having H at 173 position, but no binding of CH59 or B6. Unlike Env gp160 in DNA/SHIV-40L, which is likely to form a closed trimer, the gp150 expressed by MVA-150 might exist in more of an open confirmation and thus allowed strong binding of non-bNAbs like F106 (**Figure 2D**). Since M23 MVA-UFO produces secreted gp140 Env protein we could not test our panel of bnAbs by flow. We did however, captured the gp140 protein from the supernatant of MVA-infected cells using concavalin A (ConA) and determined binding *via* ELISA. We found that NIH 45-46 and CH58 bound well to the secreted gp140 protein followed by 3BNC117 and PGT121. PGT145, CH59, PGT151 and PG16 bound very poorly to the gp140 UFO protein (**Figure 2E**). Additionally, we characterized the UFO protein secreted by MVA and found that relative to purified gp140 UFO protein, the UFO protein secreted by the MVA construct formed mostly aggregates (**Figures 2F, G**). Overall, SHIV vaccine immunogens were shown to possess and express the proper SIV Gag and HIV Env proteins with expected sizes, and the Envs expressed by DNA and two MVA recombinants differ in their antigenicity for binding to bNAbs and non-nAbs.

### DM-150 immunogen induces higher V1V2 responses and better binding to membrane anchored envelope compared to DM-UFO

**Figure 3:**
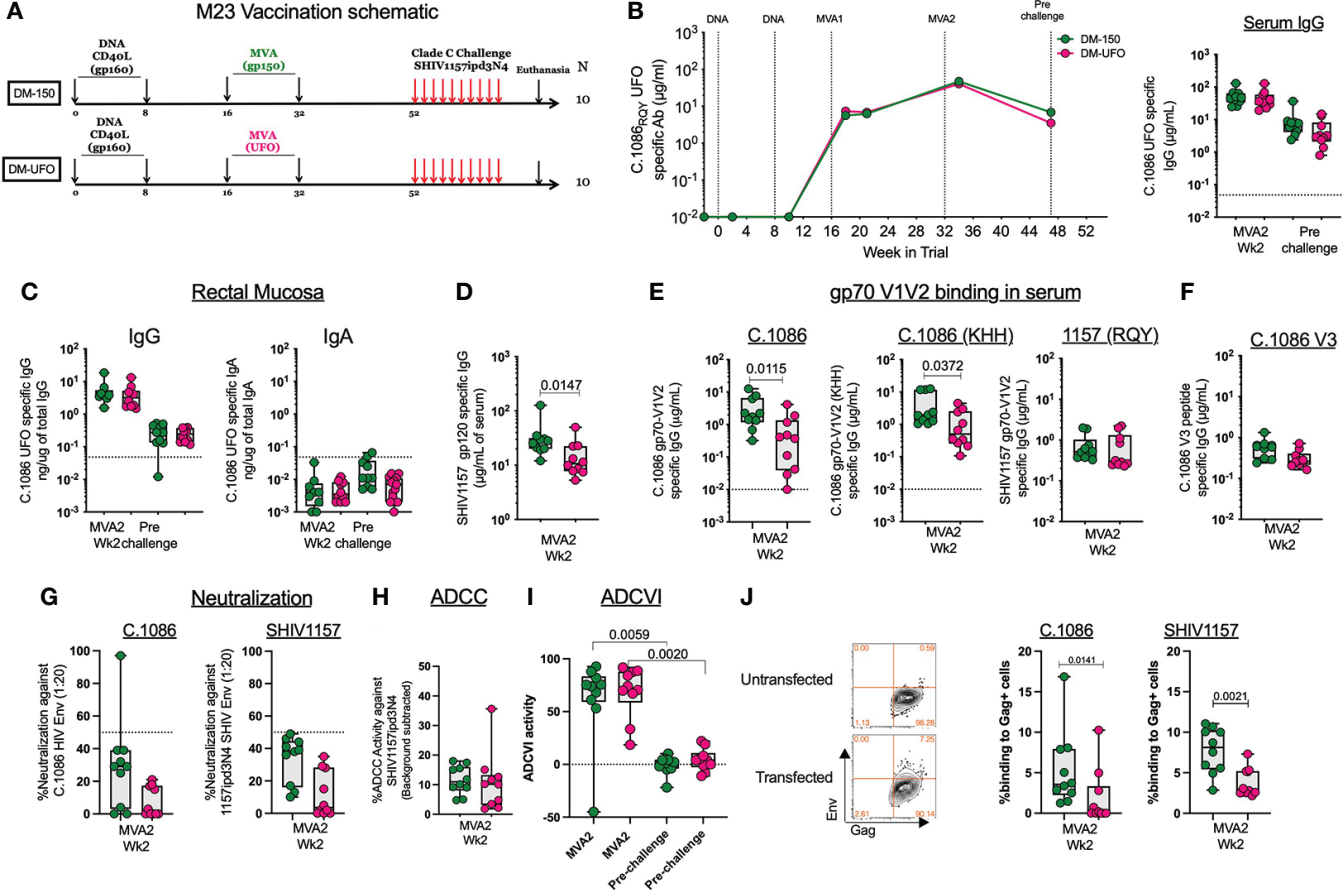
Humoral immunity following vaccination. **(A)** Schematic of the vaccine strategy. **(B)** Longitudinal serum antibody response to C.1086 gp140_UFO_ protein. **(C)** C.1086 gp140_UFO_ IgG and IgA antibody responses in the rectal secretions at the peak and prechallenge time points. **(D)** SHIV1157ipd3N4 gp120 serum binding antibody responses post MVA2. **(E)** Serum binding antibody responses to C.1086, C.1086_KHH, and SHIV1175 gp70 V1V2 constructs post MVA2. **(F)** C.1086 V3 binding antibody responses post MVA2. **(G)** Neutralization activity against C.1086 (RQY) and SHIV1157ipd3N4 pseudoviruses post MVA2. **(H)** ADCC activity against the challenge virus, SHIV1157ipd3N4, post MVA2. **(I)** ADCVI against SHIV1157ipd3N4 at the MVA2 (peak) and prechallenge time points **(J)** Binding of serum antibody post MVA2 to 293 T cells transfected with DNA/SHIV C.1086 or DNA/SHIV SHIV1157ipd3N4.

Twenty RMs were primed ID with 3mg of DNA/SHIV C.1086 (D) at wks 0 and 8 followed by booster vaccinations at wks 16 and 32 with either MVA-150 or MVA-UFO at 1x10^8^ pfu/mL (**Figure 3A**). Both MVA vaccines induced strong binding antibody response to Env UFO protein in serum with a geometric mean titer of 6.5g/ml following MVA1 and 44μg/ml following MVA2 vaccination (**Figure 3B**). These responses contracted about 8-fold over 16 weeks post MVA2 (prechallenge). Similarly, MVA vaccinations also induced Env-specific IgG in rectal secretions at 2 weeks post MVA2 with a geometric mean value of 3.7ng of specific IgG/1μg of total IgG, which contracted about 17.7-fold by prechallenge (**Figure 3C**). The total binding antibody response in the serum and rectal secretions was comparable between DM-150 and DM-UFO at all time points (**Figures 3B, C**). However, sera from DM-150 vaccinated RMs showed 2-fold higher binding to SHIV1157ipd3N4 gp120 compared to sera from DM-UFO RMs (**Figure 3D**). We also found that DM-150 vaccination induced sera showed 8.6-fold and 4.2-fold higher binding to gp70-C.1086 V1V2 scaffold proteins containing the homologous (RQY) or heterologous (KHH) V2 HS sequence respectively (**Figure 3E**). However, binding to the SHIV1157ipd3N4 challenge virus Env derived V1V2 scaffold was similar between the two groups, though lower relative to C.1086 V1V2 specific responses (**Figure 2E**). Both sera showed very low reactivity to C.1086 V3 peptide (**Figure 3F**). Functionally, both vaccine regimens elicited poor neutralizing antibody responses against the immunogen, C.1086, and challenge virus, SHIV1157ipd3N4, pseudoviruses (**Figure 3G**). ADCC activity against the challenge virus was observed, but at low level (**Figure 3H**). Additionally, ADCVI activity indicated a high level of inhibition of the challenge virus post MVA2 in both vaccine strategies, however by the time of challenge these responses waned to almost undetectable levels (**Figure 3I**). We further measured binding antibody response to the gp160 form of C.1086 and SHIV1157ipd3N4 Envs expressed on 293T cells. We observed low binding activity (0-12%) to both Envs but the binding was marginally higher in the DM-150 vaccinated RMs compared to DM-UFO vaccinated RMs (**Figure 3J**). Taken together, these data demonstrated that although DM-150 and DM-UFO vaccines induced similar overall gp140_UFO binding antibody response, sera from DM-150 vaccine animals shows higher binding activity to SHIV1157 gp120, gp70-C.1086 V1V2 scaffolds, and cell surface expressed gp160 compared to DM-UFO sera.

### Both vaccines induce comparable total SHIV (Gag+Env) specific CD4 and CD8 T cell responses

**Figure 4:**
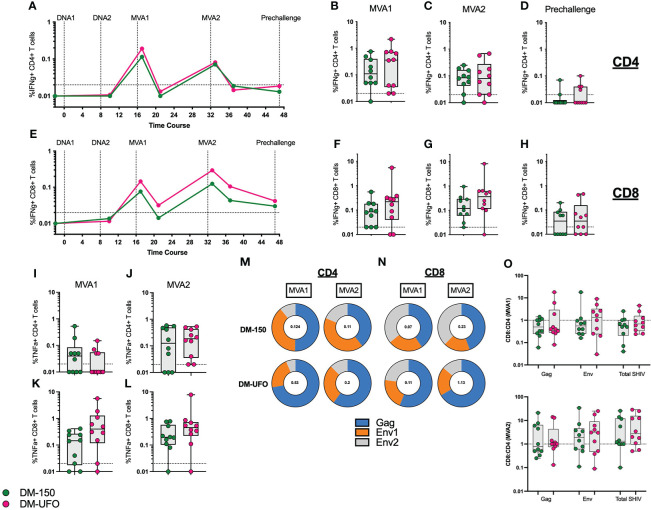
ID immunization leads to lower vaccine specific CD4 and CD8 T cell responses. **(A)** kinetics of the total SHIV specific IFNγ+ CD4 T cell response. **(B)** Total SHIV specific IFNγ+ CD4 T cell response post MVA1. **(C)** Total SHIV specific IFNγ+ CD4 T cell response post MVA2. **(D)** Total SHIV specific IFNγ+ CD4 T cell response at prechallenge. **(E)** Kinetics of the total SHIV specific IFNγ+ CD8 T cell response. **(F)** Total SHIV specific IFNγ+ CD8 T cell response post MVA1. **(G)** Total SHIV specific IFNγ+ CD8 T cell response post MVA2. **(H)** Total SHIV specific IFNγ+ CD8 T cell response at prechallenge. **(I)** Total SHIV specific TNFα+ CD4 T cell response post MVA1. **(J)** Total SHIV specific TNFα+ CD4 T cell response post MVA2. **(K)** Total SHIV specific TNFα+ CD8 T cell response post MVA1. **(L)** Total SHIV specific TNFα+ CD8 T cell response post MVA2. **(M)** Contribution of the SIV Gag, HIV Env1, and Env2 individual responses to the total IFNγ+ CD4 T cell response at MVA1 and MVA2. **(N)** Contribution of the SIV Gag, HIV Env1, and Env2 individual responses to the total IFNγ+ CD8 T cell response at MVA1 and MVA2. **(O)** Ratio of CD8 to CD4 total SHIV cytokine responses post MVA1 and MVA2. Green indicates DM-150 and magenta indicates DM-UFO.

We monitored SIV Gag and C.1086 Env specific IFNγ+ and TNFα+ CD4 and CD8 T cell responses throughout the vaccine trial. As with our previous studies (2–10, 26), we observed induction of both CD4 and CD8 T cell responses following the two MVA boosts that showed an expansion (week 1 post boost) and contraction (week 5 post boost). The IFNγ+ CD4 T cells peaked after the MVA1 vaccination with a geometric mean frequency of about 0.2% of total CD4 T cells and showed a lower recall following MVA2 vaccination (**Figures 4A–C**). In contrast, the IFNγ+ CD8 T cells peaked after MVA2 vaccination with a geometric mean frequency of 0.07% and 0.14% in DM-150 and DM-UFO groups respectively (**Figures 4E–G**). At prechallenge, the frequency of IFNγ+ CD4 T cells were mostly below the level of detection and the IFNγ+ CD8 T cells were present at low frequency in about half the animals in each group (**Figures 4D, H**). We found that there was no significant difference in the total SHIV (Gag+Env) specific CD4 or CD8 T cell response between the DM-150 and DM-UFO groups at any time point. By TNFα expression, we observed the peak CD4 T cell response 1 week post MVA2, with a geometric mean frequency of 0.09 and 0.14 for DM-150 and DM-UFO respectively. Likewise, for TNFα specific CD8 T cells, the peak was 1 week post MVA2 with a gemetric frequency of 0.18 and 0.37 for DM-150 and DM-UFO groups respectively (**Figures 4I–L**). We did however observe a predominant SIV Gag CD4 T cell response in the DM-UFO group at one week post MVA1 and MVA2 vaccinations compared to a HIV C.1086 Env dominant response in the DM-150 vaccine group (**Figure 4M**). Similar results were also seen in the total SHIV specific CD8 T cell response (**Figure 4N**). Given that these immunizations were done ID, we measured the ratio of antigen specific CD8 to CD4 T cell post MVA1 and MVA2. We found that there was a trend towards higher CD8 T cell response in the DM-UFO vaccine group relative to the DM-150 group post MVA1 for Env specific responses (**Figure 4O**). Cumulatively, these data demonstrate that the magnitude of the CD4 and CD8 T cell responses between the DM-150 and DM-UFO were similar, however, the DM-UFO vaccination induced a more SIV Gag dominant response and DM-150 vaccination induced a more Env dominant response. In addition, these data suggest that DM-UFO vaccination induced a T cell response that was more skewed towards CD8 than CD4 relative to DM-150 responses.

### ID DNA/MVA immunization induce lower magnitude of CD4 and CD8 T cell response compared to IM immunization

**Figure 5:**
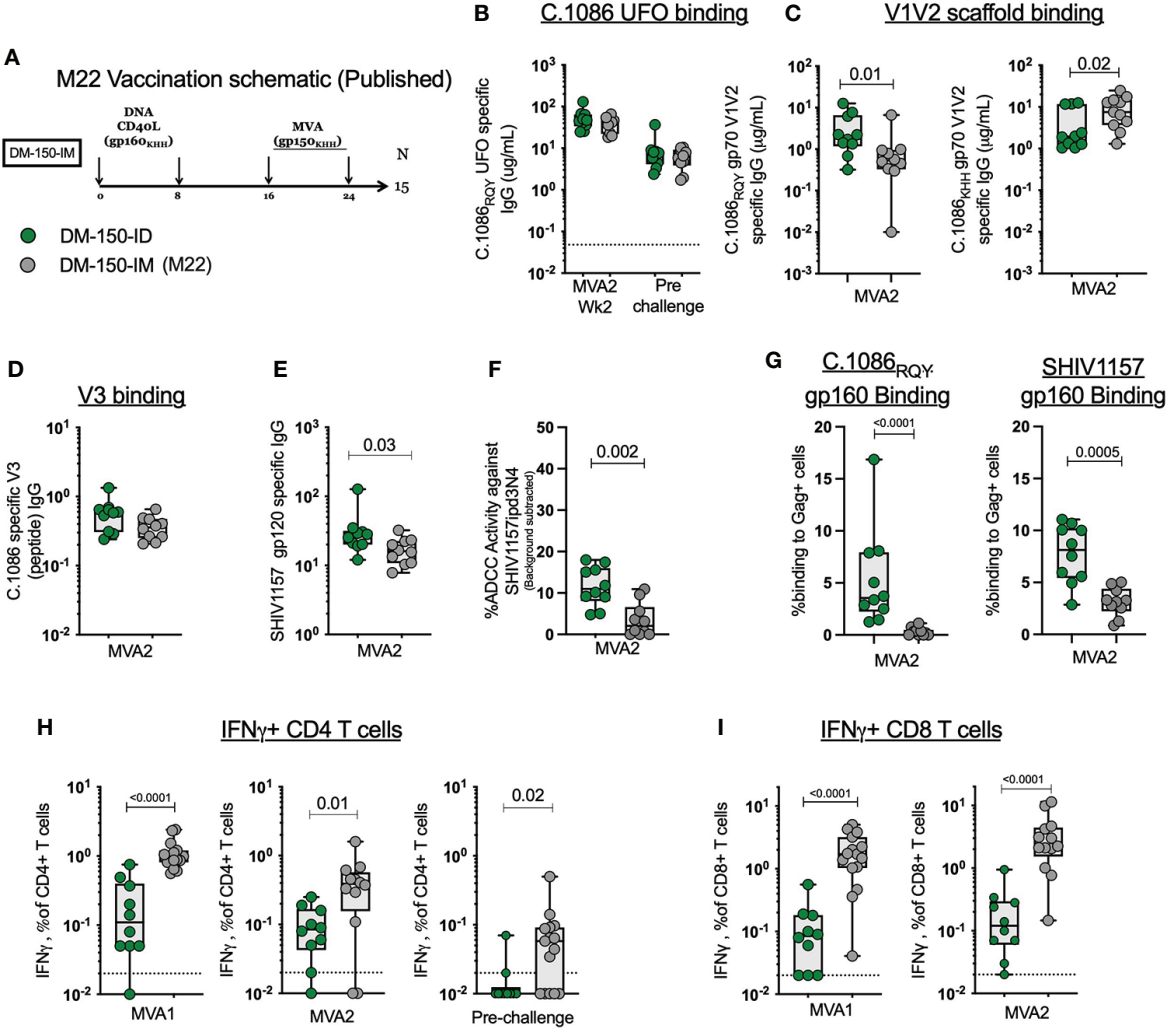
ID immunization dampens the immunogenicity of the antigen specific B and T cell response compared to IM immunization. **(A)** Published M22 vaccination schematic. **(B)** MVA2 and prechallenge binding antibody responses to C.1086_RQY_ UFO protein **(C)** C.1086 wt and mutated V2 HS gp70 V1V2 binding antibody responses. **(D)** C.1086 V3 binding antibody responses. **(E)** SHIV1157ipd3N4 gp120 binding antibody responses. **(F)** ADCC activity against the challenge virus SHIV1157ipd3N4. **(G)** Binding of serum antibody post MVA2 to 293 T cells transfected with DNA/SHIV C.1086 or DNA/SHIV SHIV1157ipd3N4. **(H)** Total SHIV specific IFNγ+ CD4 T cell response post MVA1, MVA2, and prechallenge. **(I)** Total SHIV specific IFNγ+ CD8 T cell response post MVA1 and MVA2.

We observed lower CD4 and CD8 T cell responses in this study where we used ID immunization relative to our previous studies where we used IM immunization (2, 10, 26, 44–46). To understand if ID immunizations induced a lower T cell response compared to IM immunizations, we compared the magnitude of the SHIV specific IFNγ+ CD4 and CD8 T cells to the DM-150 regimen in our previous M22 study (**Figure 5A**) **(** 2). The M22 DM-150 regimen differed from the current study in the following ways: the C.1086 gp150 protein carried the WT V2 HS (K166, H170, H173) rather than the modified V2 HS (166R, 170Q, 173Y) to match the clade C consensus sequence, immunogens were delivered IM rather than ID, and MVA1 and MVA2 were administered 8 weeks apart rather than 16 weeks apart (**Figure 4A**). When comparing DM-150_IM to DM-150_ID, we observed similar serum binding antibody response to C.1086_RQY_ UFO protein between the two routes of administration (**Figure 5B**). Antibody responses directed against the wt gp70 V1V2 (KHH) protein were significantly higher (P=0.0147) in the DM-150_IM group while the antibody response to the mutated gp70 V1V2 (RQY) was significantly higher (P=0.0197) in the DM-150_ID group (**Figure 5C**). The route of immunization had no effect on the elicitation of V3 specific binding antibody (**Figure 5D**). However, ID immunization induced significantly higher (P=0.0288) binding antibody to the challenge virus SHIV1157ipd3N4 gp120 protein (contains V2 HS matched to clade C consensus) and significantly improved (P=0.0021) ADCC activity against the challenge virus (**Figures 5E, F**). Likewise, we observed significantly higher binding to both membrane anchored C.1086 (P <0.0001) and SHIV1157ipd3N4 (P=0.0005) Envs on 293T transfected cells in ID immunized RMs compared to IM immunized RMs (**Figure 5G**). These results suggested V2 HS modification likely induced better anti-V2 and functional antibody response against SHIV1157ipd3N4.

The more profound difference between ID and IM routes of immunization was seen with the T cell response. Upon stimulation with homologous peptides pools, we found that IM immunization elicited 9-times more SHIV-specific IFNγ+ CD4 T cells post MVA1 (P<0.0001) and 3-times more at MVA2 (P=0.01) (**Figure 5H**). Similarly, IM administration elicited 17-times higher SHIV-specific IFNγ+ CD8 T cell response post MVA1 (P<0.0001) and 18.5 times more MVA2 (P<0.0001)(**Figure 5I**). Collectively, these data demonstrated that ID immunizations markedly dampen the induction of CD4 and CD8 T cell responses by DNA/MVA vaccination, while inducing comparable or better antibody response compared to IM immunizations.

### DM-UFO vaccination delays acquisition of a tier 2 SHIV1157ipd3N4 intrarectal challenge in Mamu- A*01 negative RMs

**Figure 6:**
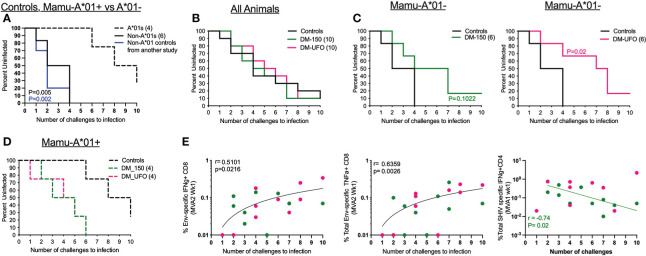
DM-UFO significantly delays acquisition of SHIV1157ipd3N4 in Mamu-A*01- RMs. **(A)** Kaplan-Meier plot depicting the number of challenges required to infect control RMs divided by Mamu-A*01 status in control animals. **(B)** Kaplan-Meier plot depicting the number of challenges required to infect all animals, including vaccinate and unvaccinated Mamu-A*01 and Mamu-A*01- RMs. **(C)** Kaplan-Meier plot depicting the number of challenges required to infect vaccinated and unvaccinated Mamu-A*01- RMs. **(D)** Kaplan-Meier plot depicting the number of challenges required to infect vaccinated and unvaccinated Mamu-A*01+ RMs. **(E)** Correlation between the number of challenges to infection and the total Env specific IFNγ+ CD8 T cell, total Env specific TNFα+ CD8 T cell, and total SHIV specific IFN γ+ CD4 T cell responses post MVA2 and MVA1.

Twenty weeks post final immunization, RMs were challenged intrarectally (i.r.) with SHIV1157ipd3N4 to determine the efficacy of our vaccine immunogens. SHIV1157ipd3N4 is a clade C tier 2 SHIV virus descended from a human clinical isolate serially passaged in infant RMs (31). Ten unvaccinated RMs were added to the study to serve as controls at the time of challenge and these RMs had no detectable T cell response to any SHIV antigens (data not shown). Vaccinated and control RMs were challenged weekly with a 1:700 dilution of the challenge stock (9.8x10^6^ TCID_50_/mL and 257 ng/mL of p27) until productive infection or for a total of 10 challenges. However, during challenges, we noticed a disproportionate infection rate in Mamu-A*01+ vs Mamu-A*01- RMs within the study (**Figure 6A**). In the control group, we found that unvaccinated Mamu-A*01+ RMs were more resistant to acquisition of SHIV1157ipd3N4 infection, while Mamu-A*01- RMs were more readily infected (**Figure 6A**). All 6 Mamu-A*01- unvaccinated animals became infected by the 4^th^ challenge with a rate of 35% per exposure. However, only 3 of the 4 Mamu-A*01+ animals became infected by the end of 10 challenges. To verify this phenomenon, we compared infection in 10 additional Mama-A*01- RMs in another study and found that all animals were infected by 4 challenges (**Figure 6A**). These data demonstrated that the rate of acquisition of SHIV1157ipd3N4 is markedly different between Mamu-A*01+ and Mamu-A*01- animals.

Because of this we analyzed protection in vaccinated animals stratified by the Mamu-A*01 status. We found no effect of vaccination in delaying the acquisition of SHIV1157ipd3N4 infection in all (A*01+ and A*01- together) animals (**Figure 6B**). However, among Mamu-A*01- animals, we observed a significant delay in acquisition of infection in the DM-UFO group compared to unvaccinated controls with a vaccine efficacy of 64% per exposure. It took 7 challenges to infect 50% of the DM-UFO animals compared to 2 challenges in the control group. This level of protection was not observed in the DM-150 group although there was a trend towards a delay in acquisition of infection with a vaccine efficacy of 57% **(** **Figure 6C**). We observed no vaccine protection among the A*01+ animals (**Figure 6D**). In fact, acquisition of infection was faster in the vaccinated Mamu-A*01+ animals compared to unvaccinated Mamu-A*01+ animals.

We performed correlations between various vaccine-induced adaptive immune parameters and the number of challenges to infection to determine which vaccine specific responses were responsible for the delay in SHIV acquisition. We found that HIV Env specific IFNγ and TNFα producing CD8 T cell responses directly correlated (p=0.02 and 0.003 respectively) with delayed SHIV acquisition in both groups, while total SHIV-specific IFNγ producing CD4 T cell responses indirectly correlated with delayed acquisition of infection but only in the DM-150 group (**Figure 6E**). Unfortunately, none of the antibody responses in serum or rectum showed any significant correlation with protection. Collectively, these data demonstrated that DM-UFO vaccination significantly delayed acquisition of SHIV1157ipd3N4 infection relative to unvaccinated Mamu-A*01- RMs, and suggested that the vaccine-induced Env-specific CD8 T cells contributed to enhanced protection while vaccine-induced Gag and Env-specific CD4 T cell responses contributed to diminished protection.

### Vaccination failed to control viral replication post infection

**Figure 7:**
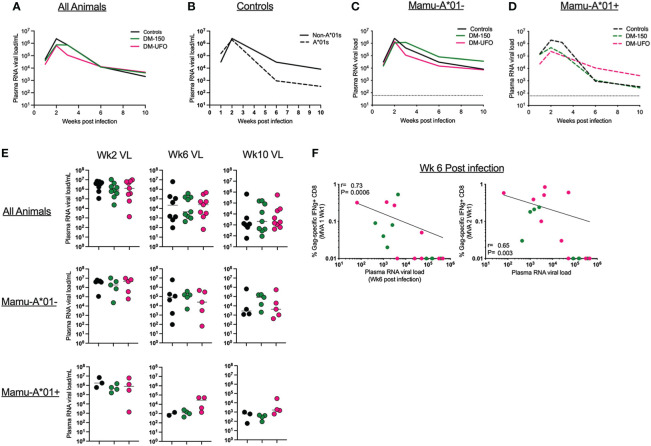
**(A)** Viral load through wk 10 post infection in all controls and vaccinated RMs. **(B)** Viral load through wk 10 post infection in controls separated by Mamu-A*01 status. **(C)** Viral load through wk 10 post infection in Mamu-A*01- RMs. **(D)** Viral load through wk 10 post infection in Mamu-A*01+ RMs. **(E)** Individual animal data of viral load at wks 2, 6, and 10 post infection. **(F)** Correlation between wk6 VL and the SIV Gag IFNγ+ CD8 T cell responses post MVA1 and MVA2.

We monitored plasma RNA viral load (VL) up to 10 weeks post infection. Overall, there was no significant difference in the plasma VL between vaccinated DM-150 and DM-UFO groups and the unvaccinated controls post infection at any time point, though there is a trend towards lower VL in the Mamu-A*01+ animals relative to Mamu-A*01- animals in the control group (**Figures 7A–E**). When divided by Mamu-A*01 status, there was no significant difference in the VL between vaccinated and unvaccinated control RMs, indicating the dichotomy seen during infection does not exist for managing virus replication (**Figures 7B–F**). We next correlated the vaccine specific CD8 T cell response with VL at wks 2, 6, and 10 post infection. We did not observe any significant correlations with peak VL at 2 wks post infection (data not shown). However, we observed a strong inverse correlation between week 6 plasma VL and SIV Gag specific CD8 T cells post MVA1 (P=0.0006) and MVA2 (P=0.003) vaccination (**Figure 7F**). Collectively, these data demonstrated that vaccination failed to have any effect on post infection viral control. This could be possibly due to low T cell response induced by ID vaccinations.

## Discussion

Here, we conducted a SHIV vaccine trial in NHPs to compare the immunogenicity and efficacy of V2 HS optimized HIV C.1086 Env immunogen presented either as a membrane anchored gp150 or a stabilized secreted gp140-UFO during MVA boost of a DNA/MVA vaccination regimen to protect against a clade C tier 2 intrarectal SHIV challenge. Our results demonstrated that while both immunogens induced comparable antibody response with few differences. Post challenge, we observed significant protection in the DM-UFO group (64% per exposure) but only in the Mamu-A*01- animals. The protection in the Mamu-A*01- animals of DM-150 (57% per exposure) was comparable to the protection in the DM-UFO group but did not reach statistical significance probably due to relatively small group sizes (n=6/group). Both vaccines failed to control virus replication in infected animals. Overall, these results demonstrated that V2 HS optimized DNA/MVA ID vaccination provides protection against intrarectal challenge in Mamu-A*01 negative animals.

Secreted gp140 expressed by a viral vector has not been well characterized in the literature. We found that MVA-UFO secreted protein was not homogenously trimeric by gel filtration and formed mostly protein aggregates by SEC. Therefore, trimers expressed by vial vector may not be comparable to trimeric proteins purified through affinity chromatography or other purification strategies. Despite not forming perfect trimers, we found the overall binding and mucosal IgG antibody responses were similar between the DM-150 and DM-UFO groups. However, when we mapped the binding responses in the serum, we found higher C.1086 V1V2 response in the DM-150 group, but similar low levels of V3 responses. We also observed similar, but low, binding antibody to the V1V2 region of the challenge virus between the two groups despite having higher overall SHIV1157ipd3N4 gp120 binding titers in the DM-150 group. Additionally, serum from DM-150 vaccinated RMs bound better to C.1086 and SHIV1157ipd3N4 gp160 VLPs. MVA-150 expresses membrane anchored gp150 and thus may present better folding and presentation of trimeric Env relative to the secreted MVA-UFO. Functionally, there was no neutralizing antibody elicited by either vaccine regimen. Collectively we can infer that the lack of complete gp41 in the DM-UFO may have contributed to lower responses against full length gp160 envelope protein relative to DM-150, however this did not affect the ability of the generated antibodies to kill cells infected with the challenge virus *via* ADCC activity.

Interestingly, we discovered a difference in the infectivity of Mamu-A*01+ and Manu-A*01- RMs by SHIV1157ipd3N4. Unvaccinated Mamu-A*01+ RMs were more resistant to infection by the challenge virus while vaccinated RMs were all infected by the 6^th^ challenge. We witnessed the reverse pattern within the Mamu-A*01- macaques. Unvaccinated Mamu-A*01- RMs were readily infected by SHIV1157ipd3N4 while vaccinated RMs were more resistant to infection by the virus. In a previous study conducted by our laboratory, the M22 trial, using the same challenge virus, we observed that all 3 animals (out of 6 Mamu-A*01+ and 9 Mamu-A*01- animals) that needed longer than 8 challenges were Mamu-A*01+ although the additional 3 Mamu-A*01+ animals were infected after the first exposure (2). None of the animals in the current study were positive for Mamu-B*08 and Mamu-B*17 alleles, which were shown to be associated with better control of SIVmac239 replication. Numerous studies have shown that Mamu-A*01+ RMs, once infected, have slower disease progression and lower set point viral loads (47–51) but there were no reports demonstrating the influence of Mamu-A*01 allele on acquisition of SIV or SHIV infection. In addition, in the current study, ID immunization resulted in low levels of both CD4 and CD8 IFNγ producing T cells (25). At this point, it is unclear what contributed to the significant delay in acquisition of infection observed in unvaccinated Mamu-A*01+ animals. A recent study showed faster acquisition of SHIV1157ipd3N4 infection in male RMs compared to female RMs, however, this study excluded Mamu-A*01+ rhesus macaques (52). Thus, we think it will be important to determine the influence of Mamu-A*01 allele on acquisition of SHIV infection using larger number of animals and to exclude Mamu-A*01+ animals for studies using SHIV1157ipd3N4 virus in rhesus macaques.

One major goal of this vaccine trial was to improve results yielded in the previous M22 vaccine trial. Three different modifications were made in the current study to improve protection. Initially, we changed the route of immunization from IM to ID and reduced the number of MVA immunizations from 3 to 2 to reduce the frequency of vaccine-induced IFNγ producing CD4 T cells that we previously showed to be negatively associated with vaccine protection by serving as potential HIV target cells in the mucosa (25). ID immunization induced low frequencies of both CD4 and CD8 T cell responses. While the lower CD4 T cell response contributed to better protection, the reduced CD8 T cell response could have resulted in lack of viral control post infection as our previous data showed that CD8 Gag responses were imperative in reducing the viral burden post infection (2). Lower CD8 T cell responses observed by ID immunization could be attributed to the different types of DCs present in the skin relative to the muscle and the ability of the vaccine to dispersed more systemically during IM immunization compared to more localized depot for ID immunization (53–55). In addition to changing the route of immunization, we also modified the V2 HS of C.1086 (K166R, H170Q, and H173Y) to mirror the V2 HS of the clade C consensus sequence. Data observed in the M22 trial, showed that vaccine elicited antibodies directed against the V2 HS were less efficient in binding to the V2 HS of the challenge virus. Upon further investigation we found that the tyrosine at position 173 was responsible for blocked binding of vaccine specific antibodies. In an effort to reverse this outcome, the V2 HS was mutated. Encouragingly, we observed improved binding to membrane anchored gp160 and ADCC activity against the challenge virus relative to the M22 trial.

Although we have gained insight on how to improve our vaccine strategy going forward, there is still work to be done. Another way to improve the current vaccine strategy would be to add a protein boost. Adding a protein boost could potentially enhance the neutralizing antibody response against the challenge virus. While giving the immunogens ID did improve vaccine specific antibody at the mucosal barrier, a protein boost could potentially heighten those responses and induce neutralizing antibodies at the site of infection. Additionally, adding a protein boost can contribute to viral control. In the M22 vaccine trial, those RMs receiving a protein boost were able to control virus replication post infection (2). Also, vaccine specific antibody responses to C.1086 gp140 as well as cross reactive SHIV1157ipd3N4 gp120 antibody responses significantly correlated with viral control. Therefore, the addition of a protein boost could serve a two-fold effect. However, selection of a protein adjuvant would be critical. The adjuvant would need to enhance the antibody response without inducing more activated CD4 T cells. In conclusion, the results from the M23 trial indicate that MVA vaccines expressing either VLP membrane-anchored or secreted C.1086 envelop induce comparable humoral and cellular immunity and provide protection against heterologous tier 2 clade C intrarectal SHIV infection in Mamu A*01- RMs.

## Data availability statement

The original contributions presented in the study are included in the article/supplementary files. Further inquiries can be directed to the corresponding author.

## Ethics statement

The animal study was reviewed and approved by Emory University Institutional animal care and use committee.

## Author contributions

TS performed the T cell assays, most of the antibody assays, analyzed the data and wrote the manuscript. PR made the DNA construct and characterized expression. SG made the MVA constructs and characterized expression. ASa designed the UFO construct and characterized expression. ASh did sample processing. SW and CD preformed neutralization experiments. PK performed ADCVI experiments and measured mucosal antibody. VV coordinated the study. RA supervised the study and wrote the manuscript. All authors contributed to the article and approved the submitted version.

## Funding

This research was supported by the National Institutes of Health grant U19 AI109633 to RA, Office of research Infrastructure Programs (ORIP/NIH) base grant P51 OD011132 to ENPRC, Emory University CFAR grant P30 AI050409.

## Conflict of interest

RA is a coinventor of the DNA/MVA vaccine technology that has been licensed to Geovax Inc. by Emory University.

The remaining authors declare that the research was conducted in the absence of any commercial or financial relationships that could be construed as a potential conflict of interest.

## Publisher’s note

All claims expressed in this article are solely those of the authors and do not necessarily represent those of their affiliated organizations, or those of the publisher, the editors and the reviewers. Any product that may be evaluated in this article, or claim that may be made by its manufacturer, is not guaranteed or endorsed by the publisher.
